# The Significance of Mycoparasitism by *Streptomyces* sp. MBCN152-1 for Its Biocontrol Activity against *Alternaria brassicicola*

**DOI:** 10.1264/jsme2.ME22048

**Published:** 2022-09-13

**Authors:** Masafumi Shimizu, Hushna Ara Naznin, Ayaka Hieno

**Affiliations:** 1 Laboratory of Plant Pathology, Faculty of Applied Biological Sciences, Gifu University, 1–1 Yanagido, Gifu city, Gifu, 501–1193, Japan; 2 River Basin Research Center, Gifu University, 1–1 Yanagido, Gifu city, Gifu, 501–1193, Japan

**Keywords:** biocontrol, mycoparasitism, endophyte, *Streptomyces*, *Alternaria brassicicola*

## Abstract

*Streptomyces* sp. strain MBCN152-1, isolated from cabbage, has potential as a biocontrol agent for *Alternaria brassicicola* on cabbage seedlings. The present study examined its mode of action. Light microscopy showed that appressorium formation by *A. brassicicola* was significantly suppressed on cabbage seedlings bacterized with MBCN152-1. Furthermore, scanning electron microscopy revealed that the mycelia of MBCN152-1, which were epiphytically growing on the cotyledon leaves of cabbage seedlings, intensively coiled around the germinating conidia of *A. brassicicola*. *In vitro* co-culture experiments demonstrated that MBCN152-1 is an aggressive mycoparasite of *A. brassicicola*, but not of *A. brassicae* or *Colletotrichum higginsianum*. Biocontrol experiments indicated that MBCN152-1 did not control diseases caused by *A. brassicae* or *C. higginsianum*. These results suggest that mycoparasitism is the primary mode of action for MBCN152-1. This is the first study to clearly demonstrate the significance of mycoparasitism in the biocontrol efficacy of endophytic *Streptomyces*.

Phytopathogenic *Alternaria* species infect a variety of plants, including vegetables, cereals, and fruit trees, and affect plant productivity, resulting in reduced quality and yields (Lou *et al.*, 2013). *Alternaria brassicicola* and *A. brassicae* are the primary causative agents of Alternaria black spot on brassica crops, such as cabbage, broccoli, cauliflower, and mustard, worldwide (Cho *et al.*, 2012; Nowicki *et al.*, 2012). They are seed-borne pathogens that infect the pods and seeds of brassica crops soon after flowering (Köhl *et al.*, 2010). According to an investigation by Kubota *et al.* (2006), 6–21% of cabbage seeds sold in the Japanese market were infested with *A. brassicicola*, and commercial cabbage seedlings grown on plug trays at nurseries were often severely damaged by this pathogen. The management of *A. brassicicola* mainly relies on the application of fungicides. However, the overuse of fungicides frequently decreases their efficacy because of the emergence of resistant strains. The development of resistance in *A. brassicicola* to major fungicides, such as iprodione, procymidone, fludioxonil, and azoxystrobin, has already been reported (Huang and Levy, 1995; Iacomi-Vasilescu *et al.*, 2004; Kreis *et al.*, 2016). In addition to fungicide resistance, food safety concerns, groundwater contamination, and environmental awareness have all prompted worldwide efforts to minimize the use of fungicides in recent years. Therefore, biocontrol using antagonistic microbes has been advocated as a viable replacement and/or augmentation to the use of conventional synthetic fungicides (Marian and Shimizu, 2019).

To date, various antagonistic fungi and bacteria, including *Chaetomium globosum*, *Trichoderma* spp., *Fusarium* sp., *Pseudomonas fluorescens*, *Serratia plymuthica*, *Bacillus amyloliquefaciens*, and *Streptomyces* spp., have been evaluated for their biocontrol potential against *A. brassicicola* (Tahvonen and Avikainen, 1987; Vannacci and Harman, 1987; Leifert *et al.*, 1993; Manhas and Kaur, 2016; Asari *et al.*, 2017). We previously screened endophytic actinomycetes isolated from surface-sterilized cabbage plants for their suppressive effects on cabbage damping-off caused by *A. brassicicola* and selected *Streptomyces* sp. strain MBCN152-1 as the best biocontrol agent (Hassan *et al.*, 2017). Symptom development following a spray inoculation with *A. brassicicola* was significantly inhibited on cabbage seedlings grown in soil mix fortified with this strain. Further studies are warranted to obtain a more detailed understanding of the mode of action of microbial biocontrol agents, which will contribute to the achievement of optimum disease control, the characterization of possible dangers to humans or the environment, and assessments of the risks of pathogen resistance development against microbial biocontrol agents (Köhl *et al.*, 2019). *Streptomyces* species are renowned for their ability to produce a number of secondary metabolites, including antimicrobial antibiotics, and this ability has been identified as the most important attribute in their biocontrol mechanisms against plant pathogens (Getha and Vikineswary, 2002; Manhas and Kaur, 2016; Vurukonda *et al.*, 2018). However, MBCN152-1 does not produce antibiotics with antifungal activity towards *A. brassicicola*.

Therefore, the present study investigated the *in planta* and *in vitro* interactions of MBCN152-1 with *A. brassicicola* to clarify its biocontrol mechanism.

## Materials and Methods

### Inoculum preparation

*Streptomyces* sp. strain MBCN152-1 was cultured on mannitol/soya agar (MSA) (Hobbs *et al.*, 1989) at 30°C for 2‍ ‍weeks. To prepare the spore suspension, colonies on the medium were suspended in 10% (v/v) glycerol solution containing 10% (v/v) dimethyl sulfoxide. The suspension was then vortexed, sonicated, and filtered through cotton to remove mycelia. Each spore suspension was adjusted to the required concentrations using a hemocytometer and was then used as the inoculum.

In the present study, *A. brassicicola* MAFF26527, *A. brassicae* O-265 (kindly provided by Prof. Hiroshi Otani, Tottori University, Japan), and *Colletotrichum higginsianum* MAFF305635 were used as the pathogens. *A. brassicicola* and *A. brassicae* were cultured at 25°C for 1‍ ‍week on potato dextrose agar and half-strength V8 juice agar containing 0.004% rose bengal (V8 juice, 100‍ ‍mL; CaCO_3_, 1.5 g; rose bengal, 0.04 g; agar, 20 g; distilled water, 900‍ ‍mL), respectively. *C. higginsianum* was cultured on potato sucrose agar at 25°C for 1‍ ‍week. The conidia of each pathogen were harvested in sterile distilled water (SDW) and filtered through two layers of sterile Kimwipes to remove mycelia. The conidial suspension of each pathogen was adjusted to the required concentrations using a hemocytometer and was then used for inoculation.

### Light microscopy of the infection behavior of *A. brassicicola* on cotyledon leaves

Cabbage seeds of cv. Matsunami were surface-sterilized with ethanol and sodium hypochlorite as previously described (Hassan *et al.*, 2017). The seeds were then bacterized with MBCN152-1 by rolling them over a 2-week-old MSA culture of the strain. These bacterized seeds were sown on 1% (w/v) water agar in a translucent polycarbonate bottle (Culture bottle CB-3; AS ONE) and incubated in an environmental control chamber (25°C, 12‍ ‍h of daylight). One week after sowing, a 20-μL aliquot of the *A. brassicicola* conidial suspension (1×10^5^ conidia mL^–1^) was spotted on the surface of each cotyledon leaf and was then incubated in the same environmental control chamber. As a control, non-bacterized seedlings were similarly inoculated with *A. brassicicola*. Conidial germination (%), germ tube length (μm), and appressorium formation (%) by the pathogen were assessed up to 48‍ ‍h post-inoculation (hpi) by microscopic observations using aniline blue staining according to previously described procedures (Hassan *et al.*, 2017). At each time point, we observed 180 conidia (30 conidia per cotyledon leaf) for each treatment. The experiment was repeated three times.

### Scanning electron microscopy (SEM) of *A. brassicicola* on cotyledon leaves of cabbage seedlings bacterized with MBCN 152-1

Cabbage seeds (cv. Matsunami) were bacterized with MBCN152-1 and then inoculated with *A. brassicicola* as previously described. As a control, non-bacterized seedlings were similarly inoculated with *A. brassicicola*. At 72 hpi, cotyledon leaves were sampled from the seedlings in culture bottles for SEM observations. These samples were fixed in 5% (v/v) glutaraldehyde (buffered to pH 7.0 with 0.1 M phosphate buffer) for 3 h. After being washed with 0.1 M phosphate buffer (pH 7.0) for 1 h, samples were fixed in 5% (v/v) osmium tetroxide (buffered to pH 7.3 with 0.1 M phosphate buffer) for 3 h, washed with 0.1 M phosphate buffer (pH 7.3) for 1 h, and dehydrated in an ethanol series (30, 50, 70, 90, and 95% for 20‍ ‍min and 100% for 20‍ ‍min×three times). Dehydrated samples were immersed in various ratios of t-butyl alcohol to ethanol (1:1, 1:2, and 0:1) for 30‍ ‍min each and then lyophilized using a t-butyl alcohol freeze-drying device (VFD-21S, VACUUM DEVICE). These specimens were attached to specimen stubs and observed with SEM (Model S-4000; Hitachi) after gold coating.

### Light and fluorescence microscopy of *in vitro* interactions between MBCN152-1 and fungal pathogens

To investigate *in vitro* interactions between MBCN152-1 and three fungal *Brassica* pathogens, namely, *A. brassicicola*, *A. brassicae*, and *C. higginsianum*, equal volumes of an MBCN152-1 spore suspension (5×10^4^ spores mL^–1^) and a conidial suspension (5×10^3^ conidia mL^–1^) of *A. brassicicola*, *A. brassicae*, or *C. higginsianum* were thoroughly mixed. A 50-μL aliquot of each mixed suspension was dropped onto the surface of a 1% (w/v) water agar plate and cultured in the dark at 27°C. Fungal pathogens cultured alone served as controls. At 3 and 8 days post-inoculation (dpi), agar blocks were excised from the inoculated points and mounted on glass slides. Fungal hyphae on the agar blocks were stained with calcofluor white MR2 (CFW; Sigma-Aldrich Chemie GmbH) according to the manufacturer’s instructions and then observed under a fluorescence microscope.

To examine the viability of *A. brassicicola* cells, fungal germ tubes and hyphae on agar blocks excised from the agar plates at 3‍ ‍dpi were stained with 0.05% Evans blue by placing 100‍ ‍μL of the solution on agar blocks, incubating for 5‍ ‍min, and then gently washing the blocks with SDW. Stained fungi on the agar blocks were observed under a light microscope.

### Assay of extracellular chitinase activity

Fifty microliters of a mixture of the MBCN 152-1 spore suspension (5×10^5^ spores mL^–1^) and the conidial suspension (5×10^4^ conidia mL^–1^) of either *A. brassicicola*, *A. brassicae*, or *C. higginsianum* were dropped onto the surface of Pridham and Gottlieb’s basal mineral salts agar medium (Pridham and Gottlieb, 1948) supplemented with 0.3% (w/v) carboxymethyl chitin (kindly provided by Prof. Shuichi Karita, Mie University, Japan), and then incubated at 27°C for 3 days. Culture plates inoculated with MBCN152-1 or *A. brassicicola* alone served as controls. The plates were stained with 0.1% (w/v) Congo red solution for 15‍ ‍min to assess chitinase activity. After the Congo red solution was discarded, 1 M NaCl solution was added to the plates and discarded 15‍ ‍min later. Chitinase activity was indicated by a clear zone (Yoshioka *et al.*, 2017).

### Biocontrol efficacy of MBCN152-1 against *A. brassicae* and *C. higginsianum* on cabbage seedlings

A spore suspension of MBCN152-1 was blended thoroughly with an autoclaved commercial soil mix (Napura, Yanmer) at a concentration of approximately 1×10^7^ spores (g soil mix)^–1^. Surface-sterilized seeds of cv. Matsunami were sown into 128-cell plug trays filled with a MBCN152-1–blended soil mix and grown in a glasshouse (23–29°C). Seven days after sowing, cabbage seedlings were spray-inoculated with a conidial suspension of *A. brassicae* (5×10^4^ conidia mL^–1^) or *C. higginsianum* (5×10^5^ conidia mL^–1^) until run-off, and were then incubated for 24‍ ‍h in a humid chamber (KCLP-1400 ICTS; NK system) maintained at >90% relative humidity and 27°C for 24‍ ‍h and then for 7 days in the aforementioned glasshouse. Seedlings grown in the untreated soil mix were also inoculated with the pathogens as controls. At the end of the experiment, the disease severity of the cabbage seedlings was evaluated according to the following scale: 0=no symptoms, 1=slightly infected (small necrotic spots formed on cotyledon leaves), 2=severely infected (large necrotic lesions formed on cotyledon leaves), and 3=dead seedlings. Each treatment comprised two replicates and each replicate used 128 plug seedlings, thereby making 256 seedlings per treatment, and the experiment was repeated three times.

## Results

### Infection behavior of *A. brassicicola* on MBCN152-1–treated cabbage seedlings

**Fig. 1.**
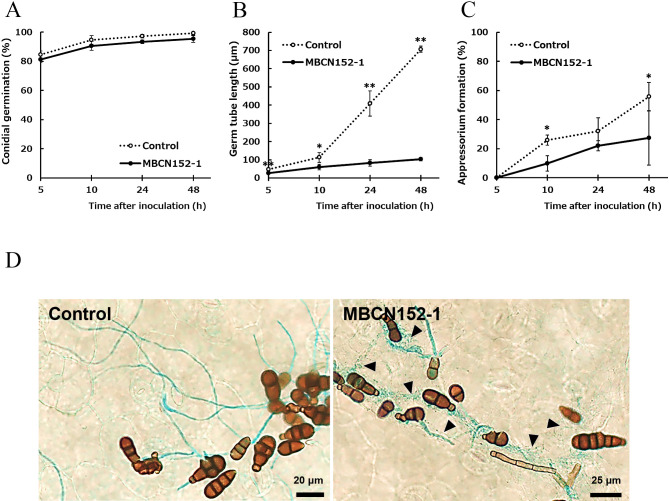
Infection behavior of *Alternaria brassicicola* on cotyledon leaves of *Streptomyces* sp. strain MBCN152-1–treated cabbage seedlings and untreated seedlings. (A) Percentage conidial germination of *A. brassicicola*. (B) Percentage appressorium formation by *A. brassicicola*. (C) Germ tube length of *A. brassicicola*. Data represent the mean±standard deviation (three replicates). * *P*<0.05; ** *P*<0.01, the Student’s *t*-test. (D) Light micrograph of *A. brassicicola* stained with 0.5% aniline blue. Black arrowheads indicate the mycelia of MBCN152-1. Observations was performed at 10 hpi.

The germination of *A. brassicicola* conidia on seedlings bacterized with MBCN152-1 was similar to that on control seedlings (Fig. 1A). However, germ tube growth and appressorium formation by the pathogen were markedly lower on bacterized seedlings than on control seedlings (Fig. 1B and C). Careful observations revealed that the majority of germinated conidia on bacterized seedlings were densely surrounded by tiny mycelia (Fig. 1D). However, these mycelia were not present around the conidia on control seedlings, indicating that those mycelia were from MBCN152-1.

### SEM of *A. brassicicola* conidia on MBCN152-1–treated cabbage seedlings

**Fig. 2.**
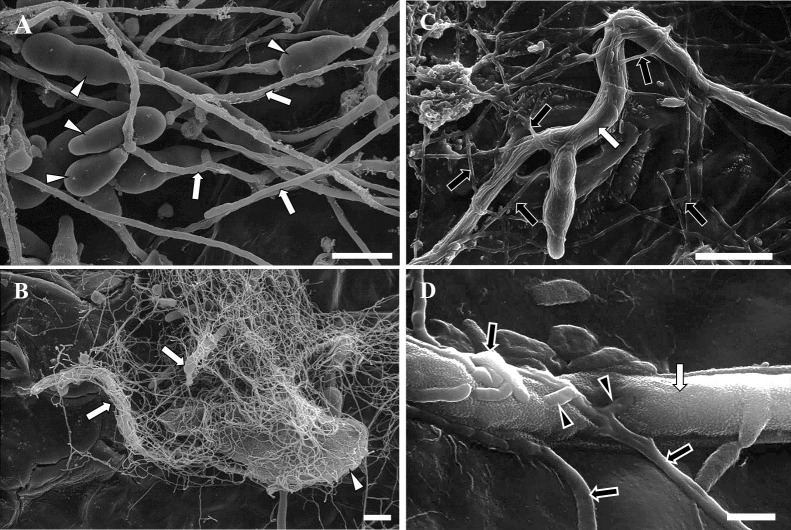
Scanning electron micrographs showing *Alternaria brassicicola* on cotyledon leaves of *Streptomyces* sp. strain MBCN152-1–treated seedlings and untreated seedlings. (A) *A. brassicicola* on the cotyledon leaves of untreated seedlings. Conidia germinated and vigorously expanded hyphae. (B) *A. brassicicola* on the cotyledon leaves of MBCN152-1–treated seedlings. The mycelia of MBCN152-1 aggregated and coiled around *A. brassicicola* germinating conidia. (C) The mycelia of MBCN152-1 clinging closely to the germ tube of *A. brassicicola*. (D) Appressorium-like structures of MBCN152-1 formed on the germ tube of *A. brassicicola*. White arrowheads and white arrows indicate the conidia and germ tubes or hyphae of *A. brassicicola*, respectively. Black arrows and black arrowheads indicate the mycelia and appressorium-like structures of MBCN152-1, respectively. Observations were performed at 72 hpi. Scale bar=10‍ ‍μm.

Using SEM, we observed *A. brassicicola* conidia inoculated onto the cotyledon leaves of control and bacterized seedlings at 72 hpi. Conidia on the control seedlings germinated and actively expanded hyphae (Fig. 2A). In contrast, conidia on bacterized seedlings also germinated; however, the growth of germ tubes and hyphae was markedly suppressed (Fig. 2B). Furthermore, germinating *A. brassicicola* conidia on bacterized seedlings were entirely covered by numerous tiny mycelia (Fig. 2B), as observed by light microscopy in the aforementioned experiment. Since these mycelia were not present on control seedlings, the mycelia covering fungal conidia were identified as MBCN152-1. The mycelia of MBCN152-1 tightly coiled around the surface of germ tubes, forming appressorium-like structures in places (Fig. 2C and D).

### Microscopic observations of *in vitro* interactions between MBCN152-1 and fungal pathogens

**Fig. 3.**
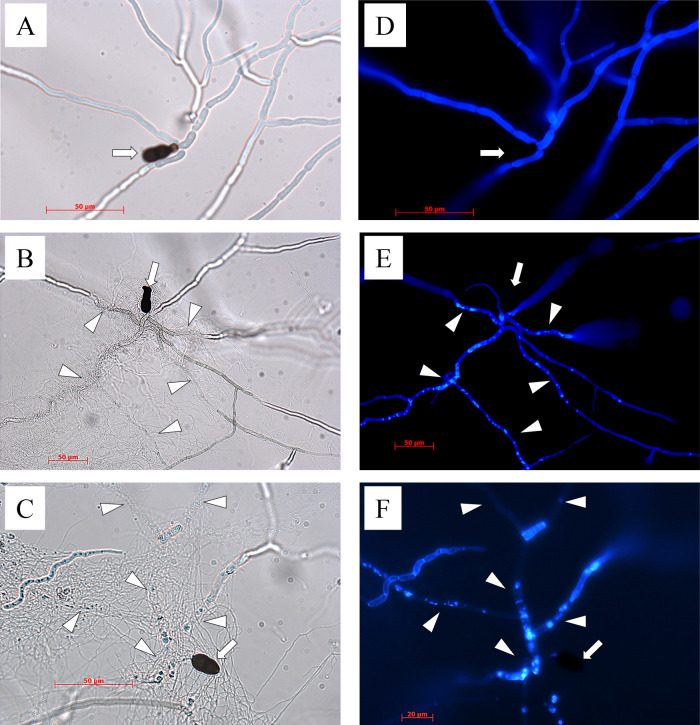
Degradation of hyphal cell walls of *Alternaria brassicicola* by *Streptomyces* sp. strain MBCN152-1. The arrows indicate the conidia of *A. brassicicola*. Substrate mycelia of MBCN152-1 multiplied around the growing germ tubes/hyphae of *A. brassicicola* (arrowheads). The hyphal cell walls of *A. brassicicola* at the interaction sites were gradually degraded. Left: light micrographs, right: fluorescent micrographs of the hyphae of *A. brassicicola* stained with calcofluor white MR2 fluorescent dye. (A and D) *A. brassicicola* was cultured alone (3 dpi). (B, C, E, and F) *A. brassicicola* was co-cultured with strain MBCN152-1. Observations were performed at 3 dpi (A, B, D, and E) and 8 dpi (C and F).

**Fig. 4.**
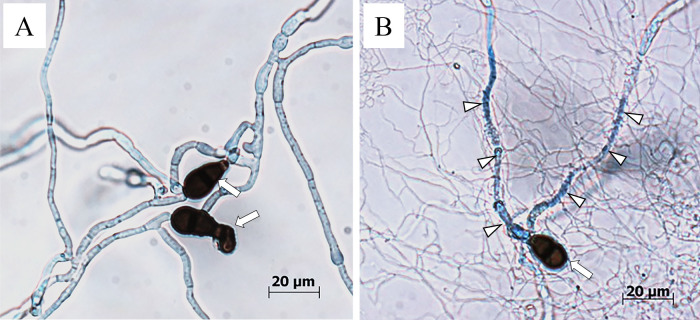
*Streptomyces* sp. strain MBCN152-1 coiled around germ tubes/hyphae of *Alternaria brassicicola* and caused cell death. (A) *A. brassicicola* cultured alone was not stained by Evans blue. (B) The germ tubes/hyphae of *A. brassicicola* coiled by MBCN152-1 were stained blue by Evans blue. Arrows indicate *A. brassicicola* conidia. Arrowheads indicate the germ tubes/hyphae of *A. brassicicola* parasitized by MBCN152-1. Observations were performed at 3 dpi.

The spores of MBCN152-1 were co-cultured with *A. brassicicola*, *A. brassicae*, or *C. higginsianum* conidia on water agar plates. Three and/or 8 dpi, the germ tubes and hyphae of fungal pathogens were stained with CFW and observed by light and fluorescence microscopy. On the control agar plate (*i.e.*, *A. brassicicola* alone), *A. brassicicola* germinated and expanded thick hyphae at 3 dpi (Fig. 3A). Conversely, when *A. brassicicola* was co-cultured with MBCN152-1, it germinated and expanded thin hyphae at 3‍ ‍dpi (Fig. 3B). On this plate, MBCN152-1 mycelia vigorously proliferated and densely coiled around the germ tubes and hyphae of *A. brassicicola*. At 8 dpi, the outline of the hyphae coiled by strain MBCN152-1 was blurred in places under the light microscope (Fig. 3C). Fluorescence microscopy of the pathogen stained with CFW revealed that many bright fluorescent spots, which were not observed in the mono-cultured pathogen, appeared in the germ tubes and hyphae coiled by MBCN152-1 at 3 dpi (Fig. 3D and E). At 8 dpi, the fluorescence of germ tubes and hyphae with a hazy outline in the region of coiling became extremely weak or largely vanished (Fig. 3F). At 3 dpi, the staining of *A. brassicicola* with Evans blue, which stains dead cells blue, revealed that the germ tubes and hyphae densely coiled by MBCN152-1 displayed intense blue staining, whereas those on the control plate exhibited faint blue staining (Fig. 4).

**Fig. 5.**
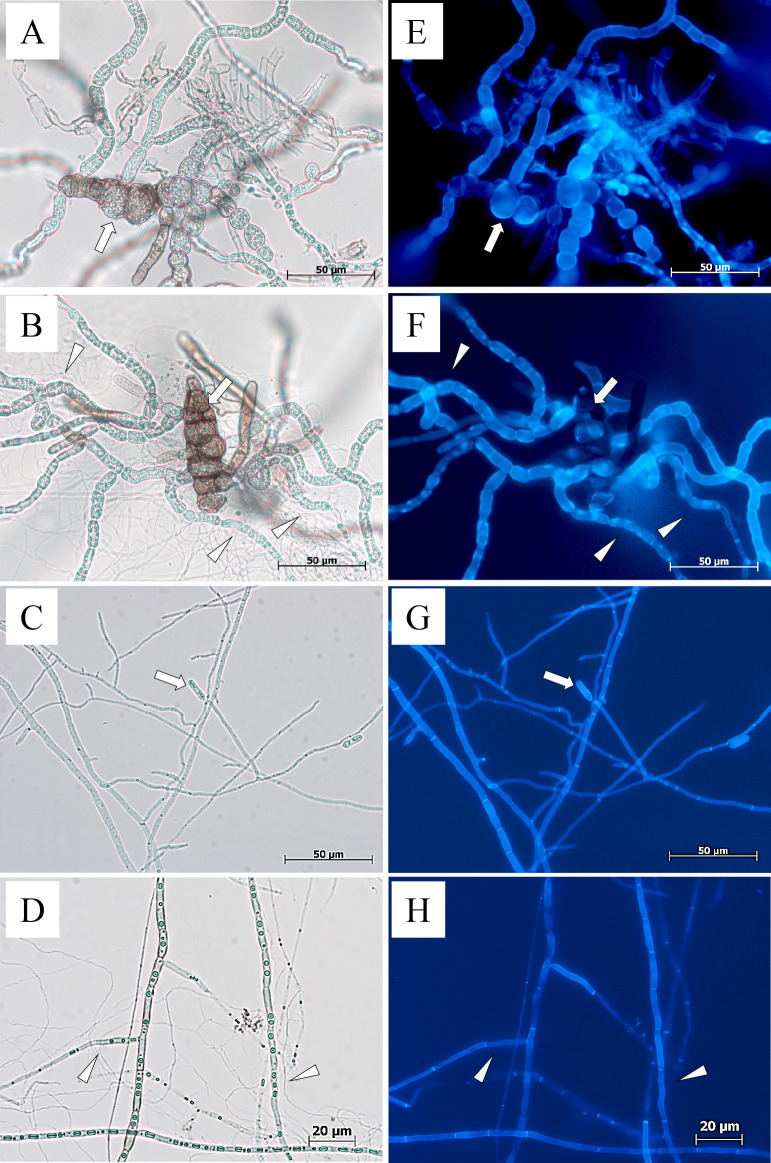
Microscopy of *Alternaria brassicicae* and *Colletotrichum higginsianum* co-cultured with *Streptomyces* sp. strain MBCN152-1 on water agar. (A and E) *A. brassicae* cultured alone. (B and F) *A. brassicae* co-cultured with MBCN152-1. (C and G) *C. higginsianum* cultured alone. (D and H) *C. higginsianum* co-cultured with MBCN152-1. Arrows indicate fungal conidia. Although MBCN152-1 expanded substrate mycelia around the germ tubes/hyphae of *A. brassicae* and *C. higginsianum* (arrowheads), fungal cell walls were not degraded. Left: light micrographs, right: fluorescent micrographs of fungal hyphae stained with calcofluor white MR2. Observations were performed at 8 dpi.

When *A. brassicae* conidia were co-cultured with MBCN152-1, they germinated and expanded hyphae, similar to mono-cultured conidia (Fig. 5A and B). Similarly, the presence of MBCN152-1 had no influence on the conidial germination or hyphal development of *C. higginsianum* (Fig. 5C and D). Furthermore, MBCN152-1 did not coil around the fungal germ tubes or hyphae of either pathogen. CFW staining showed that the cell walls of germ tubes and hyphae of both fungi were not damaged by their interactions with MBCN152-1 (Fig. 5E, F, G, and H).

### Induction of chitinase production by MBCN152-1 following a co-culture with *A. brassicicola*

When cultured alone, *A. brassicicola* did not produce chitinase (Fig. S1A), whereas MBCN152-1 exhibited weak chitinase activity on chitin-amended agar medium (Fig. S1B). Conversely, when MBCN152-1 was co-cultured with *A. brassicicola*, it exhibited stronger enzymatic activity than when the strain was cultured alone (Fig. S1C). In contrast, the chitinase activity of MBCN152-1 did not increase when co-cultured with *A. brassicae* and *C. higginsianum* (Fig. S1D and E). These results indicated that the presence of *A.‍ ‍brassicicola* stimulated chitinase production by MBCN152-1.

### Biocontrol effects of MBCN152-1 on *A. brassicae* and *C. higginsianum* on cabbage seedlings

Cabbage seedlings grown on soil mix fortified with MBCN152-1 were spray-inoculated with* A. brassicae* or *C. higginsianum*. The severity of disease symptoms caused by either pathogen was not reduced by MBCN152-1 (Table 1), indicating that the strain did not exert any biocontrol effects on these fungi.

## Discussion

In recent years, many researchers have been investigating the potential use of endophytic actinomycetes as biocontrol agents against plant diseases because they are among the predominant taxa of the endophytic microbial communities of many plants and are considered to participate in plant natural defenses against plant pathogens (Shimizu, 2011; Cao *et al.*, 2020; Marian *et al.*, 2020; Zhuang *et al.*, 2020). We previously reported that endophytic *Streptomyces* sp. strain MBCN152-1, which is closely related to *S. humidus*, exhibited excellent biocontrol activity against cabbage damping-off caused by *A. brassicicola* (Hassan *et al.*, 2017). However, the mechanisms by which MBCN152-1 controls *A. brassicicola* remain unclear. Deciphering the mode of action of MBCN152-1 is crucial for unlocking its full biocontrol potential. Therefore, the present study investigated the biocontrol mechanism of MBCN152-1 and discovered that mycoparasitism of the strain plays a key role in its biocontrol activity.

Many *Streptomyces* strains have been assumed to employ antibiosis mediated by antimicrobial antibiotics as one of the primary mechanisms of their biocontrol of plant pathogens (Getha and Vikineswary, 2002; Vurukonda *et al.*, 2018). Manhas and Kaur (2016) demonstrated that biocontrol strain *S. hydrogenans* DH16 suppressed conidial germination and hyphal growth by *A. brassicicola* via antibiosis, resulting in a reduction in damping-off and black leaf spots on radish seedlings. However, MBCN152-1 lacks the ability to produce antifungal antibiotics that inhibit the growth of *A. brassicicola* (Hassan *et al.*, 2017), indicating that mechanisms other than antibiosis are involved in its biocontrol activity. To examine the influence of MBCN152-1 on infection by *A. brassicicola*, we microscopically examined the behavior of the fungus on the surface of cabbage seedlings. We found that conidial germination was not significantly different, which was consistent with previous findings (Hassan *et al.*, 2017); however, germ tube elongation and appressorium formation were significantly suppressed on seedlings bacterized with MBCN152-1. We also reported that MBCN152-1 mycelia, which were epiphytically growing on the surface of cotyledon leaves, coiled around and formed appressorium-like structures on the germinating conidia of *A. brassicicola*. Coiling and the formation of appressorium-like structures is a parasitic behavior that is typical of mycoparasitic fungi, such as *Trichoderma* spp. and *Clonostachys rosea* (Tu, 1988; Tapio and Pohto-Lahdenperä, 1991; Anees *et al.*, 2010; Hasan *et al.*, 2022). Several mycoparasitic *Streptomyces* strains have also been reported to parasitize fungal pathogens in similar man­ners‍ ‍(Tu, 1988; Palaniyandi *et al.*, 2013). Therefore, we hypothesized that the epiphytic mycelia of MBCN152-1 parasitized germinating *A. brassicicola* conidia and directly suppressed the pre-infection growth of germ tubes and appressorium formation. Previous studies demonstrated that some *Streptomyces* spp., such as *S. albus* (Tu, 1986), *S.‍ ‍griseoviridis* (Tapio and Pohto-Lahdenperä, 1991), *S.‍ ‍alni*‍ ‍(Ziedan *et al.*, 2010), *S. phaeopurpureus* (Palaniyandi *et al.*, 2013), and *S. plicatus* (Chen *et al.*, 2016), exhibit mycoparasitic activity against fungal pathogens. However, mycoparasitism by *S. humidus* or its related species on phytopathogenic fungi has not been reported.

In the present study, we further validated the mycoparasitic activity of MBCN152-1 against *A. brassicicola* using *in vitro* co-culture experiments. When MBCN152-1 was co-cultured with *A. brassicicola* on water agar, it intensively coiled and proliferated around fungal germ tubes and hyphae by 3 dpi, similar to that observed on cotyledon leaves. Furthermore, Evans blue staining demonstrated that fungal cells densely coiled by the strain were dead at 3 dpi. Fluorescence microscopy using CFW staining revealed several bright fluorescent spots on fungal cells in the regions of coiling, implying that MBCN152-1 mycelia affected the cell wall structure of the pathogen. This result coincides with a previous study by Elad *et al.* (1983), who detected similar intense fluorescence on fungal hyphae parasitized by *Trichoderma harzianum* using CFW staining. They concluded that this fluorescence was a sign of the localized lysis of host cell walls by *Trichoderma*. Therefore, MBCN152-1 appeared to induce the partial lysis of the cell walls of *A. brassicicola* germ tubes and hyphae, possibly by penetrating these fungal cells from appressorium-like structures, and killed them within 3 days of contact. Cell wall lysis by MBCN152-1 became more pronounced at 8 dpi. The fluorescence of fungal cell walls stained with CFW had largely vanished in the coiling zones. Cell wall lysis during mycoparasitism is mediated by a set of hydrolytic enzymes, such as chitinases and β-1,3-glucanase (Gruber and Seidl-Seiboth, 2012). *Streptomyces* spp. are well-known prolific producers of a number of cell wall-degrading enzymes, including chitinases, glucanases, and peptidases (Joo, 2005; Fróes *et al.*, 2012; Jiang *et al.*, 2012; Lv *et al.*, 2021). When MBCN152-1 was cultured on chitin-amended medium, it also produced chitinase. Furthermore, we discovered that when the strain was co-cultured with *A. brassicicola* on the‍ ‍same medium, it secreted a large amount of chitinase. On the other hand, when MBCN152-1 was co-cultured with‍ ‍two other fungal pathogens (*A. brassicae* and *C. higginsianum*), its production of chitinase did not increase. These results suggest that MBCN152-1 recognizes *A. brassicicola* as its prey, actively secretes chitinase to attack and lyse the fungal cell wall, and feeds on dead cell contents. In *Trichoderma* species, the production of cell wall-degrading enzymes was induced prior to and during contact with the host fungus during mycoparasitism (Gruber and Seidl-Seiboth, 2012). The activation of mycoparasitism-related processes, including cell wall-degrading enzyme secretion, occurs in *Trichoderma* spp. after host-derived external signals are perceived or host-derived ligands bind to receptors (Steyaert *et al.*, 2003; Alfiky and Weisskopf, 2021). The mechanisms by which MBCN152-1 recognizes *A. brassicicola* and initiates its mycoparasitic behavior is an extremely interesting topic for future research.

To clarify the role of mycoparasitism in the mode of action of MBCN152-1, we assessed its mycoparasitic activity against two other *Brassica* pathogens, namely, *A. brassicae* and *C. higginsianum*, and found that it was not parasitic to either pathogen. This result contradicted previous findings by Tapio and Pohto-Lahdenperä (1991), who reported that mycoparasitic *S. griseoviridis* strain K61, which was originally isolated from Finnish Sphagnum peat as a biocontrol agent against *A. brassicicola*, parasitized *A. brassicicola* and several other fungal pathogens, such as *Botrytis cinerea*, *Sclerotinia sclerotiorum*, and *Fusarium oxysporum*. Therefore, MBCN152-1 was found to have a limited host range, in contrast to strain K61. Furthermore, the results of biocontrol experiments demonstrated that MBCN152-1 was incapable of controlling diseases caused by *A. brassicae* and *C. higginsianum*. Therefore, given the concordance between mycoparasitic activity and disease control efficacy, we concluded that mycoparasitism is the primary biocontrol mechanism of MBCN152-1.

In summary, the present results demonstrated that epiphytically colonized mycelia of *Streptomyces* sp. MBCN152-1 act as a trap net on the surface of cabbage seedlings, preferentially parasitizing and killing the germinating conidia of *A. brassicicola* during the pre-infection growth stage, consequently preventing the damping-off of seedlings. To the best of our knowledge, this is the first study to clearly demonstrate the importance of mycoparasitism by endophytic *Streptomyces* species for its biocontrol activity.

## Citation

Shimizu, M., Naznin, H. A., and Hieno, A. (2022) The Significance of Mycoparasitism by *Streptomyces* sp. MBCN152-1 for Its Biocontrol Activity against *Alternaria brassicicola*. *Microbes Environ ***37**: ME22048.

https://doi.org/10.1264/jsme2.ME22048

## Supplementary Material

Supplementary Material

## Figures and Tables

**Table 1.** Biocontrol effects of *Streptomyces* sp. strain MBCN152-1 against *Alternaria brassicae* and *Colletotrichum higginsianum* on cabbage seedlings

	Disease severity	
	*Alternaria brassicae*	*Colletotrichum higginsianum*
Control	1.66±0.36	0.97±0.08
MBCN152-1	1.52±0.43	1.00±0.06

Data represent the mean±standard deviation (three repeated experiments). Statistical ana­lyses were performed with the Mann–Whitney U test.
