# Supplementary material for: The Significance of Mycoparasitism by Streptomyces sp. MBCN152-1 for Its Biocontrol Activity against Alternaria brassicicola
Source: Microbes Environ. 2022 Sep 13;37(3):ME22048. doi: 10.1264/jsme2.ME22048 (PMC9530718; doi:10.1264/jsme2.ME22048)
Supplement: Supplementary file 1 — Supplementary Material [file 37_22048_s1.pdf]

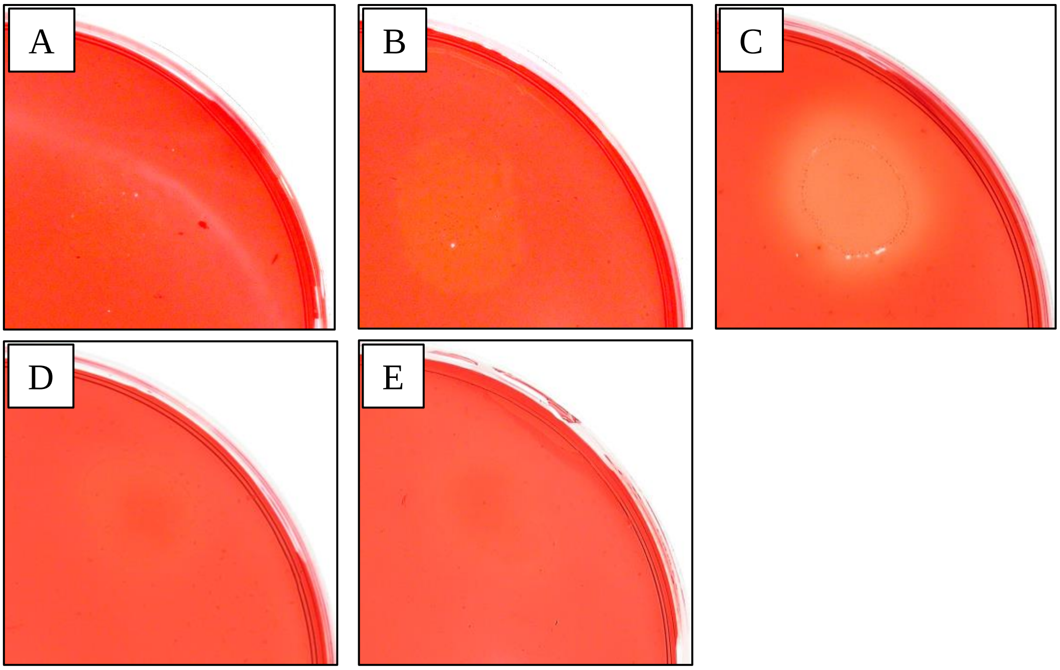

Fig. S1 Induction of chitinase in *Streptomyces* sp. strain MBCN152-1 by co-culturing with *Alternaria brassicicola* on CMC-ISP9 agar. (A) *A. brassicicola* was cultured alone. (B) MBCN152-1 was cultured alone. (C) MBCN152-1 was co-cultured with *A. brassicicola*. (D) MBCN152-1 was co-cultured with *A. brassicae*. (E) MBCN152-1 was co-cultured with *C. higginsianum*. Spore suspensions (50 μl) of either individual microbe or mixture of both microbes were drop-inoculated onto plate. Chitinase activity was visualized by staining with 0.1% Congo red followed by de-staining with 1 M NaCl.
